# Management of dermatologic toxicities related to epidermal growth factor receptor inhibitor therapy across Europe: can we get a consensus?

**DOI:** 10.3332/ecancer.2011.220

**Published:** 2011-10-13

**Authors:** L Lemmens

**Affiliations:** Department of Gastroenterology, University Hospitals Leuven, Leuven, Belgium

## Abstract

To date, there are no phase III trial data that can guide healthcare professionals in managing toxicities of epidermal growth factor receptor inhibitors (EGFRIs). This exploratory survey assessed the similarities and differences in nursing management of EGFRI-related toxicities across 10 European countries. A questionnaire that was sent to ten nurses who specialize in the treatment of patients with EGFRI asked about the prevention and treatment of acneiform rash, dry skin/eczema, fissures, paronychia, and pyogenic granuloma. Responses from seven nurses showed that overall (with the exception of rash), treatment differed markedly between countries in the management of dermatologic toxicities. These substantial differences across the European hospitals surveyed suggest that it might be worthwhile to develop treatment algorithms by conducting a consensus conference or a follow-up survey with several assessments and a larger sample.

Cancer treatment is being transformed with the advent of entirely new classes of drugs that specifically inhibit genes, proteins, or processes involved in cancer development, thus sparing a greater number of healthy cells. One cancer drug target is the epidermal growth factor receptor (EGFR, also called ErbB1 and human epidermal growth factor receptor 1 (HER 1)). EGFR is a member of a protein family called receptor tyrosine kinases, which control vital cell processes such as proliferation, migration, and survival [1]. EGFR has been linked to many common cancers, including head and neck cancer, colorectal cancer (CRC), pancreatic cancer, and non-small-cell lung cancer (NSCLC), and overproduction of EGFR is associated with more aggressive clinical behavior (including more tumor angiogenesis, proliferation, and metastasis) and shorter overall survival time [2–4]. Based on their efficacy in clinical trials, therapies that inhibit the EGFR signaling pathway, such as the small-molecule inhibitors, erlotinib (Tarceva^®^) and gefitinib (Iressa^®^), and the monoclonal antibodies, cetuximab (Erbitux^®^) and panitumumab (Vectibix^®^), are now routinely used in clinical practice to control these cancers [5–8].

Several types of epithelial cells, such as those in the epidermis, hair follicles, sebaceous glands, mucosal tissues, and ocular tissues, produce high levels of EGFR. Epidermal growth factor receptor inhibitors (EGFRIs) are thus commonly associated with class-specific toxicities, many of which are new to oncology teams and differ from the toxicities regularly observed with conventional chemotherapy regimens [9]. Skin toxicities, such as acneiform rash, dry skin, fissures, and nail and hair disorders, are commonly associated with treatment with EGFRIs [10,11]. To date, there are no data from phase III trials that can guide healthcare professionals in managing toxicities of EGFRIs.

In this article, a first step towards improving and standardizing patient care is reported. In brief, an exploratory survey of nurses from different European countries was conducted to assess the similarities and differences in the management of EGFRI-related toxicities across European countries. Between April and November 2009, a questionnaire was sent to ten hospital-based clinical nurse specialists who focus on the treatment of patients with EGFRI; these nurses were selected based on their prior participation in a nursing advisory board on skin toxicities of EGFRIs or membership in the European Oncology Nursing Society TARGET task force on targeted therapies. The questionnaire asked about the prevention and treatment of EGFRI-related dermatologic toxicities including acneiform rash, dry skin/eczema, fissures, nail changes such as paronychia, and pyogenic granuloma. All questions were open-ended to allow for the possibility of discovering management approaches that are not reported in the literature. Seven nurses responded, from Belgium, Ireland, the Netherlands, Spain, Sweden, Switzerland and the United Kingdom. As shown in the tables and discussed in detail below, approaches to management of EGFRI toxicities differed substantially across the nurses surveyed.

## Skin rash

All survey respondents provided patient education and general skin care advice, including the use of moisturizers (Table 1), but they also reported that they do not provide routine pharmaceutical prophylaxis for skin rash. In some countries, topical antibiotic creams are given immediately with the appearance of grade 1 acneiform rash, while in others oral antibiotics are preferred. Treatment of grade 3 acneiform rash varies greatly, with oral/topical steroids as well as antibiotics and antihistamines being incorporated into some strategies. Overall, there appeared to be substantial variation in the management of skin toxicities across Europe. These results are consistent with another survey that inquired about the management of EGFRI-associated skin rash across 110 oncology practices in the United States, which also detected pronounced diversity in the types of interventions used [12].

An interdisciplinary, international panel has recommended dividing papulopustular reactions into three categories, based on the severity of symptoms, the impact of symptoms on daily activities, and whether the patient has signs of, or potential for, superinfection [13,14]. According to the international guidelines, if there is no response to rash treatment within 2—4 weeks, EGFRI therapy should be temporarily suspended as directed by the manufacturer [13,14]. Therapy can be restarted once the rash adequately diminishes in severity. Referral to a dermatologist is warranted if lesions have an uncharacteristic appearance or distribution; if there is necrosis, blistering, or petechial/purpuric lesions; or if patients have atypical dermatological manifestations unrelated to rash [15]. However, consistent with the findings of this survey, all recommendations are based on experience rather than clinical trial data, and there are important geographic variations in actual management of EGFRI skin toxicity [11].

## Dry skin

Various approaches to preventing dry skin were recommended, but the use of emollients and protection against the sun were among the most common (Table 2). Management approaches differed from each other with regard to the types of emollients to be used; whether and when to use corticosteroids; and the need for, and timing of, referral to a dermatologist. Not all respondents mentioned examining patients for the possibility of infection, although it could be assumed that these evaluations are conducted when infections are suspected.

## Fissures

For fissures, there was virtually no overlap in the therapeutic measures reported, although two respondents reported using propylene glycol for grade 1 fissures. Four respondents reported giving pain medication, but the timing of its initiation varied. Only two respondents reported experience with grade 4 fissures. In one of these centers reduced dose or interrupted EGFRI therapy for severe fissures. Common recommendations for the treatment of fissures in Europe include a 50% solution of propylene glycol in water, an ointment containing 10% salicylic acid, or a hydrocolloid dressing or liquid cyanoacrylate glue [11]. Patient education also differed widely among the respondents, according to whether prophylactic care is recommended for the entire body, just the hands and feet, just the hands, or just the feet (see Table 3).

## Paronychia

Treatment of paronychia among the respondents varied even more markedly than treatment of the other dermatologic toxicities (see Table 4). For grade 1 paronychia, management strategies ranged from observation to the use of an oral antibiotic. Most respondents gave some sort of topical treatment for grade 1; two added a corticosteroid for grade 3. Antibiotic therapy was given if needed. Three respondents considered referral to a dermatologist for moderate or severe paronychia. Only two respondents reported experience with grade 4 paronychia; EGFRI therapy was routinely stopped for grade 4 paronychia at one of the centers.

## Pyogenic granuloma

Only three respondents reported experience with any grade of pyogenic granulomas because this condition is rarely observed (see Table 5). One nurse felt that no effective treatment was available for this condition. For grade 1 pyogenic granulomas, the three respondents recommended various antiseptic soaks; they recommended adding topical treatment and oral antibiotics (if superinfections were present) for grade 2. A short course of nonsteroidal anti-inflammatory drugs was also suggested to alleviate pain. For prophylactic care, three nurses advised patients to moisturize their bodies twice daily with an alcohol-free emollient and to minimize exposure to the sun or use broad-spectrum sun protection. One nurse also recommended that patients take short showers without soap.

## Conclusion

This pilot survey of European hospitals revealed that there is no consensus on the management of EGFRI-related skin toxicities, but respondents clearly agreed that these toxicities need treatment. This is not surprising, given that treatment options described in the medical literature are based on case reports, case series, or reported personal experience, not on data from large, randomized, controlled trials. Other potential explanations for differences in management are that (a) the specific approved EGFRIs and their indications vary between countries in Europe and (b) different agents have slightly different toxicity profiles.

Despite the diversity in their answers, the respondents clearly recognized that EGFRI toxicities are treatable and require medical intervention. While dermatologic reactions are usually mild to moderate in severity, they are sometimes physically or emotionally bothersome such that patients wish to discontinue therapy. Conversely, patients may under-report their symptoms out of fear that the anticancer therapy will be withheld. Therefore, it is important that healthcare professionals minimize the incidence and severity of side effects, prevent their progression, and thus the need for dose reduction or discontinuation of potentially effective treatment.

Standardized consensus guidelines to prevent or treat EGFRI-related dermatologic toxicities may help improve patient care and be a useful resource for clinicians throughout Europe. These guidelines should include detailed information on how to educate patients about lifestyle modifications, early signs of toxicity, and the importance of treatment adherence; how to regularly assess patients’ side effects and related quality of life; and how to provide resources for psychosocial support. Assessment or patient self-report of side effects should begin within 14 days from EGFRI initiation [7].

This survey was exploratory, and the results should be interpreted in the context of certain limitations. The survey is not representative of the entire European Union or of all hospitals within each country. A more detailed follow-up survey with an expanded questionnaire is planned in order to facilitate the development of a consensus statement and, potentially, algorithms for the optimal treatment of toxicities. In the meantime, management of EGFRI-related toxicities should be undertaken on a case-by-case basis, with an emphasis on preventing dose modification whenever possible. Effective management and patient education may help to alleviate the significant social and emotional anxiety related to these manageable toxicities, thus resulting in improved quality of life.

## Figures and Tables

**Table 1:** Prevention and treatment of acneiform rash

**Country**	**Prevention/prophylaxis**	**Grade 1**	**Grade 2**	**Grade 3**	**Grade 4**	**Other treatment**
Belgium	Patient education; skin care starter set (if available from EGFRI manufacturer).	Use skin cleansing; moisturizing cream; further self-care instruction; if needed, oral minocycline 100 mg/day in evening.	As for grade 1, except give minocycline 200 mg/day in evening, depending on patient need and severity of rash.	As for grade 1, except give 200 mg minocycline. For superinfection, add other antibiotics. For pruritus, add analgesic and/or antihistamine.	Not seen yet.	No response.
Ireland	Use moisturizing cream (water-based) on limbs, hands, and feet. Avoid sun (wear protective clothes). Use sunblock SPF 35.	Use metronidazole cream twice daily.	As for grade 1, except add minocycline 100 mg/day. For itching, add antihistamine.	Use metronidazole cream up to 5 times daily, minocycline 100 mg/day (increased to 200 mg/day if required), saline compresses 15 min twice daily. For itching, add antihistamine. For *S. aureus* superinfection, add broad-spectrum antibiotics. If no response, consider dose-adjusting or interrupting EGFRI.	No guidelines.	No response.
The Netherlands	Moisturize skin twice daily with a neutral cream on the entire body. Use sun protection; take short showers, no soap.	Use metronidazole cream 1%–2% 100 mg twice daily. If no improvement after 2 weeks, increase to 200 mg.	As for grade 1, with or without additional oral steroids.	As for grade 1, plus oral steroids. If no improvement after 2 weeks, EGFRI dose delay or reduction.	Not seen yet. Grade 4 is rare.	No response.
Spain	Advise patients to moisturize dry areas of the body twice a day (e.g. using a thick alcohol-free emollient). Minimize exposure to sunlight and use broad-spectrum sunscreen (SPF > 15).	Use topical hydrocortisone (1% or 2.5%) and clindamycin 1% gel.	As for grade 1, plus tetracycline oral antibiotics and oral antihistamine for itch.	As for grade 2. Consider involving dermatologist.	As for grade 3. Consider intravenous antibiotic and a culture to determine bacterial strain if antibiotic resistance is suspected.	Avoid acne vulgaris medications as these treatments could irritate and worsen the skin rash.
Sweden	Patient education, written guidelines.	Employ local treatment, take systemic treatment into consideration.	Employ local and/or systemic treatment.	Employ systemic treatment. Consider EGFRI dose reduction or interruption.	Employ systemic treatment. Refer to skin specialist. Interrupt EGFRI.	There is a special list of creams, ointments, and drugs that must be used with different grades.
Switzerland	Usually no prophylactics are used. Provide patient education. Use skin care starter set (from EGFRI manufacturer); content is optimized according to hospital practice.	Phase I: Cleanse skin, use moisturizing cream, give further instruction for self-care. Phase II: Possibly begin antibiotics, depending on patient need and severity of rash. Phase III: Begin antibiotic, often clindamycin; corticosteroid cream depending on patient need and severity of rash.			Not seen yet.	No response.
United Kingdom	None.	Use topical clindamycin.	Add oral antibiotic, ‘Wyamycin’ or minocycline.	Dose-reduce EGFRI; hydroxyzine; dermal lotion and ‘Betnovate’ scalp application.	Stop EGFRI.	No response.

EGFRI, epidermal growth factor receptor inhibitor; SPF, sun protection factor.

**Table 2:** Prevention and treatment of dry skin/eczema

**Country**	**Prevention/prophylaxis**	**Grade 1**	**Grade 2**	**Grade 3**	**Grade 4**	**Other treatment**
Belgium	Instruct patients to use moisturizers without high fat content.	Use emollients.	Use strong emollients. For dry eczema, use corticosteroid cream (1–2 weeks). For wet, superinfected eczema, use topical fusidic acid + antibiotics (5–10 days).	Use emollients. Some short use of steroids/antibiotics in case of infection.	Not seen yet.	No response.
Ireland	Use mild soaps, avoid harsh laundry detergents, use tepid water when showering or bathing.	Use emollients. For eczema, use weak topical corticosteroids, ‘Modrasone’ ointment.	As for grade 1; if superinfected, add antibiotics.	Not seen yet.	Not seen yet.	No response.
The Netherlands	Moisturize skin twice daily with a neutral cream on the entire body. Use sun protection; take short showers, no soap.	Use 20% white paraffin in cetomacrogol cream.	Use an oral antihistamine (hydroxyzine or cetirizine); for eczema, intermittent corticosteroid: fluticasone cream 0.5 mg/g (‘Cutivate’) or betamethasone cream 1 mg/g (‘Betnelan’) 7–14 days.		Not seen yet.	No response.
Spain	Advise patients to moisturize dry areas of the body twice a day (e.g. using a thick alcohol-free emollient). Minimize exposure to sunlight and use broad-spectrum sunscreen (SPF > 15).	Use topical hydrocortisone (1% or 2.5%) and clindamycin 1% gel.	As for grade 1, plus tetracycline oral antibiotics and oral antihistamine for itch. Consider involving dermatologist.	Not seen yet.	Not seen yet.	No response
Sweden	Provide patient education, adhere to written guidelines.	Observe if asymptomatic, use steroid creams if symptomatic.	As for grade 1. Use ‘soft creams.’	As for grades 1 and 2. Employ local treatment.	As for grade 3. Consult skin specialist.	There is a special list of creams, ointments, and drugs that must be used with different grades.
Switzerland	Instruct patient to use moisturizers without high fat content.	Use emollients with urea.	As for grade 1.	As for grade 1. Some use of steroids if dermatologist suggests.	Not seen yet.	No response.
United Kingdom	None.	Use ‘Diprobase’.	Use ‘Oilatum’ bath salts and lotion.	Refer to dermatologist.	Refer to dermatologist.	‘E45 cream’.

EGFRI, epidermal growth factor receptor inhibitor; SPF, sun protection factor.

**Table 3:** Prevention and treatment of fissures

**Country**	**Prevention/prophylaxis**	**Grade 1**	**Grade 2**	**Grade 3**	**Grade 4**	**Other treatment**
Belgium	Use emollient cream several times a day (after hand wash). Take off shoes.	Use hand cream, ‘Fucidin’ crème, cover fissures in evening.	Use hand cream. For open fissures, ‘Fucidin.’ Use propylene glycol 50% aqueous solution under plastic occlusion (30 min/day), salicylic acid 10% ointment, hydrocolloid dressing.	Use emollients with urea. Cover open fissures with propylene glycol three times daily, after that use emollients or ‘Fucidin’ with bandage. Use systemic pain medication if necessary.	Not seen yet.	Use of fine cotton gloves while working and during night time. Patients are very inventive; they experiment with lots of different creams and bandages.
Ireland	Avoid tight shoes.	Use propylene glycol 40% in aqueous cream with 2% salicylic acid twice daily.			Not seen yet.	No response.
The Netherlands	Moisturize skin twice daily with a neutral cream on the entire body. Use sun protection, take short showers, no soap.	Use 50% propylene glycol in cetomacrogol cream.	As for grade 1; use paracetamol or ibuprofen.	Not seen yet.	Not seen yet.	No response.
Spain	Keeps hands dry and out of water as much as possible, or use gloves.	Consider topical antiseptics such as topical silver and topical hydrocortisone (1% or 2.5%) and clindamycin 1% gel.	Consider involving dermatologist.	Not seen yet.	Not seen yet.	To reduce pain, redness and swelling, consider a short course of an NSAID (e.g. 400 mg ibuprofen 2–3 times/day).
Sweden	Provide patient education, adhere to written guidelines.	Use ‘Alsolsprit’ compress.	As for grade 1 plus ‘Betnovate’ with chinoform if needed.	As for grade 2 plus culture and oral antibiotics.	As for grade 3.	No response.
Switzerland	Use emollient cream twice daily on hands and feet.	Use emollients with urea; examine painful areas for open wound.	Use emollients with urea, protect open fissures with ‘Fingerrisse’ bandage or according to patient preference.	As for grade 2.	Not seen yet.	Use fine cotton gloves while working.
United Kingdom	None.	Use ‘DiproBase’ cream.	Pyridoxine.	Increase dose of pyridoxine and reduce EGFRI dose.	Stop EGFRI drug.	Use antibiotic ointments.

EGFRI, epidermal growth factor receptor inhibitor; NSAID, nonsteroidal anti-inflammatory drug.

**Table 4:** Prevention and treatment of paronychia

**Country**	**Prevention/prophylaxis**	**Grade 1**	**Grade 2**	**Grade 3**	**Grade 4**	**Other treatment**
Belgium	Educate patient that this may occur.	No response.	Rinse with local antiseptic; individualized decision for antibiotics. Protect fingertips according to patient preference.		Not seen yet.	No response.
Ireland	No response.	Use antiseptic soaks twice a day (e.g. one part vinegar to ten parts water for 5 min each time).	As for grade 1 plus ‘Lotriderm’ cream. For superinfection, give broad-spectrum antibiotics. If skin is oozing, take a swab.	As for grade 2, plus NSAIDs to control pain.	Not seen yet.	No response.
The Netherlands	Moisturize skin twice daily with a neutral cream on the entire body. Use sun protection; take short showers, no soap.	Use silver nitrate applicators (75% silver nitrate, 25% potassium nitrate) once daily.	As for grade 1, plus high-class corticosteroid (clobetasol cream 0.5 mg/g (‘Dermovate’), minocycline, or doxycycline 100 mg/day). For infection, give flucloxacillin 3 × 500 mg/day for 7 days.		Not seen yet.	No response.
Spain	Keeps hands dry and out of water as much as possible, or use gloves. Avoid too-tight shoes, to prevent friction on the nail.	Try frequent applications of topical petrolatum around nails. Consider topical antiseptic and/or topical antibiotic.	Try topical steroid 2–3 times/day. Oral antibiotic must be considered if a bacterial infection is suspected. Consider culturing and involve dermatologist.	Not seen yet.	Not seen yet.	To reduce pain, consider a short course of an NSAID (e.g. 400 mg ibuprofen 2–3 times/day).
Sweden	Provide patient education, adhere to written guidelines.	Use ‘Alsolsprit’ compress.	Cultures; with infection, give oral antibiotics.	As for grade 2; consider referral to skin specialist.	As for grade 3.	No response.
Switzerland	Educate patient that this may occur.	No therapy is needed in the absence of lesions.	Rinse with local antiseptic; individualized decision for antibiotics. Protect fingertips according to patient preference.		Not seen yet.	No response.
United Kingdom	None.	Give oral antibiotic—flucloxacillin.	Add penicillin V.	Refer to dermatologist.	Stop EGFRI.	No response.

EGFRI, epidermal growth factor receptor inhibitor; NSAID, nonsteroidal anti-inflammatory drug.

**Table 5:** Prevention and treatment of pyogenic granuloma

**Country**	**Prevention/Prophylaxis**	**Grade 1**	**Grade 2**	**Grade 3**	**Grade 4**	**Other Treatment**
Belgium	Patient education that this may occur.	No ‘effective’ treatment. Use antiseptic (chloramine, povidon iodine), NSAID to control pain, drying paste and antiseptic (chlorhexidine), or silver nitrate weekly. For superinfection, use oral antibiotics. Partial or total nail extraction may be necessary.			No response.	No response.
Ireland	No response.	Use antiseptic soaks twice a day (e.g. one part vinegar to ten parts water for 5 min each time).	As for grade 1 plus betamethasone diproprionate/ clotrimazole cream. For superinfection, use broad-spectrum antibiotics. If skin is oozing, take a swab.	As for grade 2, plus NSAIDs to control pain.	Not seen yet.	No response.
The Netherlands	Moisturize skin twice daily with a neutral cream on the entire body. Use sun protection. Take short showers without soap.	No response.			No response.	No response.
Spain	Advise patients to moisturize dry areas of the body twice a day (e.g. thick alcohol-free emollient). Minimize exposure to sunlight and use broad-spectrum sunscreen (SPF > 15).	Use a topical antiseptic such as topical silver nitrate weekly.	Oral antibiotics combined with topical treatment. Consider involving dermatologist and/or surgeon.	Not seen yet.	Not seen yet	To reduce pain, consider a short course of an NSAID (e.g. 400 mg 2–3 times/day).
Sweden	No response.					
Switzerland	No response.		Not seen yet.	No response.		
United Kingdom	No response.		Not seen yet.	No response.		

EGFRI, epidermal growth factor receptor inhibitor; NSAID, nonsteroidal anti-inflammatory drug; SPF, sun protection factor.
